# Effects of phytosterols' intake on systemic and tissue-specific lipid metabolism in C57BL/6J mice

**DOI:** 10.3389/fnut.2022.924236

**Published:** 2022-07-27

**Authors:** Qian Zhu, Jingjing Wu, Jianling Li, Shengquan Wang, Daxue He, Xuemei Lian

**Affiliations:** ^1^Key Laboratory of Molecular Biology for Infectious Diseases, Ministry of Education, Center for Lipid Research, Chongqing Medical University, Chongqing, China; ^2^Department of Nutrition and Food Hygiene, School of Public Health and Management, Chongqing Medical University, Chongqing, China; ^3^Qiannan Center for Disease Control and Prevention, Duyun, China

**Keywords:** phytosterols, cholesterol, lipid metabolism, tissue specificity, RNA-seq

## Abstract

This study aimed to investigate the long-term effects of phytosterols (PS) intake on systemic and tissue-specific lipid metabolism in C57BL/6J mice. Healthy male C57BL/6J mice were randomly divided into control diet group (CS) and PS diet group (2% PS). After 28 weeks of continuous feeding, serums, livers, and lungs were collected for targeted free sterols quantification, biochemical tests, lipid profile detection, and RNA-seq analysis. Compared with the CS group, 2% PS supplementation significantly increased campesterol concentrations and its ratio to cholesterol in the serum, liver, and lung of mice, with cholestanol concentrations and its ratio to cholesterol decreased. Total cholesterol (TC) levels were reduced in the serum of the PS group (*p* < 0.05), with the triglyceride (TG) levels unchanged. In response to the decreased circulating cholesterol concentration, the expression of endogenous cholesterol synthesis genes was upregulated in the liver, but caused no obvious lipid accumulation and inflammatory cell infiltration. However, for peripheral tissues, long-term PS-fed mice exhibited diminished cholesterol synthesis, fatty acid transport, and oxidation in the lung. The results provided clear indication that 2% PS diet effectively reduced circulating TC levels in the healthy mice, with tissue-specific lipid metabolic regulation in the liver and the lung.

## Introduction

Phytosterols (plant sterols/stanols) (PS) are natural dietary ingredients, mainly derived from vegetable oils, cereals, legumes, nuts, fruits, and vegetables. Because they share structural similarities with cholesterol, it is well-established that PS interfere with intestinal cholesterol absorption and get the systemic cholesterol-lowering effect (1–3). The European Atherosclerosis Society recommended daily intake of 2 g PS is effective in reducing low-density lipoprotein (LDL)-cholesterol levels by 8–10% (4).

In the early days of PS research, it is believed that PS can hardly appear in the circulation. At present, increasing evidence suggests that even though the absorption rate of PS is low compared to cholesterol, it does appear in the circulation and plasma concentrations increase after higher intake. The small amounts of PS absorbed are rapidly taken up by the liver and transported to different tissues by lipoproteins (4). However, the evidence related to pathophysiological effect of absorbed PS on tissue-specific metabolic and functional markers is still scarce.

The liver is the central organ of lipid metabolism and responds positively to the dynamic state of circulating cholesterol. Even though beneficial effects of PS on diminishing high-fat diet-induced liver inflammation were reported recently (5), parenteral nutrition (PN) with a soy-based lipid emulsions (SOLE), which are rich in plant sterols, increase the risk for cholestasis and hepatobiliary injury was also observed (6). This paradox warrants further study.

The lung is not a classical organ suffering from lipid disturbance, while current studies clearly indicate that cholesterol is necessary to maintain the normal physiological function of type II alveolar cells (7–9). The dynamic balance of alveolar lipids is closely related to the immune response (10–12) and even cancer environment (13, 14) in the lung. Therefore, emerging roles have connected lipid metabolism with lung physiology and immune homeostasis, and this intriguing area of research holds the promise of uncovering novel lipid-regulating treatments for controlling lung diseases. Studies by our group and others demonstrated the protective role of PS on LPS-induced acute lung injury in mice (15) and patients with asthma (16). Still, it is an emerging frontier in the field of pulmonary research and fundamental studies are needed.

To demonstrate tissue-specific lipid metabolism and its possible pathophysiological effect, 2% PS were added to the diet of healthy C57BL/6J mice in this study. After 28-week feeding, the PS supplementation increased free sterol levels in multiple tissues, including serum, liver, and lung. With the decrease in circulating cholesterol concentration, the expression of endogenous cholesterol synthesis genes was upregulated in the liver, but caused no obvious lipid accumulation and liver inflammation. In contrast, genes associated with cholesterol synthesis, fatty acid transport, and oxidation were downregulated in peripheral tissues like the lung. Part of these results was published as a conference abstract in the American Society for Nutrition meeting, NUTRITION 2021 LIVE ONLINE (17).

## Materials and methods

### Animals and diets

All animal experiment procedures were in accordance with the principles of medical ethics, reviewed and approved by the Biomedical Ethics Committee of Chongqing Medical University (Chongqing, China) and carried out in accordance with the approval guidelines (approval document no. 2021008). The protocols for both laboratory animal handling and experiments were in strict accordance with the Chongqing Management Approach of Laboratory Animal (Chongqing government order no. 195). Six-week-old healthy male wild-type C57BL/6J mice (*n* = 24) were purchased from the Experimental Animal Center of Chongqing Medical University and housed in a specific pathogen-free animal laboratory (constant temperature and humidity, 12 h light/dark cycles, with adequate sterile food and drinking water). Body weight of the mice was measured weekly. The animals were randomly divided into control diet group (CS) and PS diet group (2% PS). The 2% PS level we used in this study is a dosage commonly used in animal studies since it reflects a ratio compared to dietary cholesterol intake seen in humans (18, 19). The PS (44.23% β-sitosterol, 25.95% campesterol, 19% stigmasterol, and others) were purchased from Xi'an Hysf Biotechnology Co., Ltd., Xi 'an, China, and the feed was synthesized by Jiangsu Madisen Biomedical Co., Ltd., Jiangsu, China. Feed formula was adjusted according to relevant literature (20, 21), and the specific formula is shown in Table 1. After adaptive feeding for 3 weeks, mice were continuously fed for 25 weeks before sacrifice.

**Table 1:** Formulation of control and phytosterols-supplemented diets for C57BL/6J mice.

**Ingredient^a^**	**Control**	**Phytosterol**
Casein	200	200
DL-Methionine	3	3
Corn starch	398	398
Maltodextrin	132	132
Sucrose	120	120
Cellulose	50	50
Soybean oil	25	25
Lard	20	20
Mineral mix AIN93G	24	24
Vitamin mix AIN93G	14	14
Choline bitartrate	1	1
Potassium citrate	10	10
Sodium chloride	2	2
Phytosterols^b^	0	20
Total	999	1019

a The feed was synthesized by Jiangsu Madisen Biomedical Co., Ltd., Jiangsu, China.

b The phytosterol was purchased from Xi'an Hysf Biotechnology Co., Ltd., Xi'an, China. Composition (as is basis): total sterols>95%; β-sitosterol, 44.23%; campesterol, 25.95%; stigmasterol, 19% and others.

### Sample collection

Animal surgery was performed under 1% sodium pentobarbital anesthesia (0.1 mg/10 g weight), and all efforts were made to minimize animal suffering. The blood samples were taken by cardiac puncture, placed for 4 h at room temperature before centrifugation, and performed at 3,000 rpm for 15 min at 4°C. The upper layer of clarification solution was collected and stored frozen at −80°C. Livers and lungs were quickly collected, weighed, and partially fixed in 4% paraformaldehyde for analyses. Others were stored at −80°C for RNA and protein extraction.

### Lipid profile detection

Serum biochemical indexes were determined using Mindray BS-400 automatic biochemical analyzer (Shenzhen, China), including aspartate aminotransferase (AST), alanine aminotransferase (ALT), total cholesterol (TC), triglyceride (TG), high-density lipoprotein (HDL)-cholesterol, and LDL-cholesterol levels. Non-HDL-cholesterol level was calculated according to the following equation:


(1) $$\begin{array}{r} \text{Non\_HDL\_Cholesterol = TC}-\text{HDL}\_\text{Cholesterol} \end{array}$$

The detection and analysis of free sterols in the serum, liver, and lung were commissioned by the Shanghai Metabolome Institute-Wuhan, Wuhan 430075, China. Tissue samples (20 mg) were extracted with 1,000 μl pre-cooled methanol (Thermo Fisher, USA) using tissue lyser at 50 Hz for 90 s (10 μl serum emulsion samples were mixed with 100 μl of pre-cooled methanol for protein precipitation). The supernatant was obtained after centrifugation at 12,000 rpm for 10 min at 4°C with the above extraction repeated once. The supernatant was dried and mixed with 200 μl pyridine solution containing 8 mg picolinic acid (Sigma-Aldrich, USA), 22 mg 2-methyl-6-nitrobenzoic anhydride (Sigma-Aldrich, USA), and 4 mg 4-dimethylaminopyridine (Sigma-Aldrich, USA), and then incubated at 80°C for 60 min. The mixture was cooled down to the ambient temperature and added with 400 μl hexane (Thermo Fisher, USA)/ethyl acetate (SCRC,China) (1:2, v/v). The extraction was dried and re-dissolved in 200 μl acetonitrile (Thermo Fisher, USA) followed by filtering the solution by a 0.22-μm membrane filter. All samples were tested on an ultra-performance liquid chromatography-mass spectrometry (1290-6470 UPLC-MS/MS, Agilent, USA). Peak determination and peak area integration were performed with MassHunter Workstation software (Agilent, version B.08.00). The concentration of free sterols was detected by external standard method. Standard curves were constructed by least-squares linear regression analysis using the peak area ratio of derivatized individual standard against the nominal concentration of the calibrator. Based on the corresponding standard curve and the peak height or peak area of the sample to be measured, the concentration of measured free sterols in the sample can be obtained.

According to instruction of the manufacturer, the TC and TG levels in the liver and lung tissues were determined using tissue TC and TG enzymatic assay kit (E1015 and E1013, respectively) from Applygen Technologies Inc., Beijing, China.

### Hematoxylin and eosin staining

The liver tissue specimens were fixed with 4% paraformaldehyde, embedded with liquid paraffin, and sectionalized with 4 μm thickness for hematoxylin and eosin (H&E) staining. The slices were roasted overnight at 60°C, dewaxed in xylene, fully hydrated with gradient alcohol at a concentration of 100–95–80–75%, washed with PBS three times, soaked in hematoxylin dye for 3–5 min, rinsed with tap water, observed the degree of nuclear staining under a microscope, adjusted the depth of nuclear staining with 1% hydrochloric acid ethanol, and stained with eosin dye solution for 1–3 min at room temperature. Finally, the slices were dehydrated and sealed with neutral resin. Images were captured with the digital biopsy scanner (Pannoramic DESK; Hungary).

### Western blot

A total of 50 mg liver tissues were harvested and the whole proteins were extracted using a mixture of 100 μl lysis buffer, 1 μl protease inhibitor (100× ), 1 μl phosphatase inhibitors (100× ), and 1 μl phenylmethanesulfonyl fluoride (100× ). Protein samples were resuspended in SDS-PAGE sample loading buffer (Beyotime Biotechnology, China) and loaded onto 6 or 8% sodium dodecyl sulfate–polyacrylamide gel electrophoresis (Bio-Rad, Hercules, CA) under denaturalizing conditions. After protein separation, it was electro-blotted onto a polyvinylidene difluoride membrane (Bio-Rad, Hercules, CA, USA), blocked with 5% skim milk powder, followed by overnight immunoblotting at 4°C with diluted primary antibody. The hybridization bands were combined with the corresponding secondary antibodies (Zhongshan, Beijing, China) conjugated with horseradish enzyme at 37°C. Protein concentration was detected *via* commercially available BeyoECL Star ultra-sensitive ECL chemiluminescence kits (Beyotime Biotechnology, China). Protein expression was quantitated using ImageJ software (NIH, Bethesda, MD, USA). The primary antibodies used in the study were β-actin (1:1,000, TA-09, Zhongshan, Beijing, China), anti-HMGCR (1:1,000, ab174830, Abcam, UK), and anti-SREBP2 (1:500, sc-13552, Santa Cruz Biotechnology, USA).

### Enzyme-linked immunosorbent assay

The concentrations of TLR4 and ICAM2 in serum and liver were determined by ELISA according to the protocol of the kit (Quanzhou Jiubang Biotechnology, Fujian, China).

### RNA-seq

Frozen liver and lung tissue samples (~50 mg) were taken for RNA-seq gene expression quantification. Total RNA was extracted from tissues using Trizol (Takara, Cat# 9109). RNA integrity was assessed using the RNA Nano 6000 Assay Kit of the Bioanalyzer 2100 system (Agilent Technologies, CA, USA). Following quality control, Illumina sequencing was carried out by pooling different libraries according to the requirements of effective concentration and target disembarkation data volume and 150 bp paired-end reads were generated. All samples were paired-end sequenced on the Illumina NovaSeq 6000 platform at Novogene Bioinformatics Technology Co., Ltd., Beijing, China. The datasets discussed in this study have been deposited in National Center for Biotechnology Information Search database's Gene Expression Omnibus and are accessible through GEO Series accession number GSE183242 (https://www.ncbi.nlm.nih.gov/geo/query/acc.cgi?acc=GSE183242). To validate the consistency of gene expression, the relative mRNA levels of gene were determined by real-time qPCR using SYBR Green qPCR master mix (MCE, HY-K053). Assays were performed in triplicate with endogenous β-actin expression as the standard control. The primer sequences are provided in Table 2.

**Table 2:** The primer sequences of RT-qPCR.

**Gene**	**Forward primer**	**Reverse primer**
*Actin*	CCACCATGTACCCAGGCATT	CAGCTCAGTAACAGTCCGCC
*Acadvl*	ACTACTGTGCTTCAGGGACAA	GCAAAGGACTTCGATTCTGCC
*Acsl1*	TCTTGGTGTACTACTACGACGAT	CGAGAACCTAAACAAGGACCATT
*Acsl3*	CTGCACAGGCGTGTTTTATGT	ACGTGGGACCAAAGAGACTAT
*Cd40*	GCAGTGTGTTACGTGCAGTG	TGTGCAGTGGCTTGTCAGTC
*Cdh1*	CAGGTCTCCTCATGGCTTTGC	CTTCCGAAAAGAAGGCTGTCC
*Cpt1a*	TGGCATCATCACTGGTGTGTT	GTCTAGGGTCCGATTGATCTTTG
*Cyp51*	TGACAGGAGGCAACTTGCTTT	GGCGAGACGGAACAGGTAG
*Fdps*	GGAGGTCCTAGAGTACAATGCC	AAGCCTGGAGCAGTTCTACAC
*Hmgcs1*	TGAACTGGGTCGAATCCAG	CCTGTAGGTCTGGCATTTCCT
*Hmgcr*	GGTCCTTGTTCACGCTCATAGTC	TCTGCTTGTAGTCTCTGCTTCCA
*Icam2*	ATGGTCCGAGAAGCAGATAGT	TGCTGTTGAACGTGGCTGT
*Il1b*	GAAATGCCACCTTTTGACAGTG	TGGATGCTCTCATCAGGACAG
*Il6*	GCCTTCTTGGGACTGATGCT	TGCCATTGCACAACTCTTTTCT
*Pparα*	AACATCGAGTGTCGAATATGTGG	CCGAATAGTTCGCCGAAAGAA
*Scd1*	TTCTTGCGATACACTCTGGTGC	CGGGATTGAATGTTCTTGTCGT
*Sqle*	AGTTCGCTGCCTTCTCGGATA	GCTCCTGTTAATGTCGTTTCTGA
*Srebf2*	GTTGACCACGCTGAAGACAGA	CACCAGGGTTGGCACTTGAA
*Tlr4*	GCCTTTCAGGGAATTAAGCTCC	GATCAACCGATGGACGTGTAAA
*TNFα*	AGGCACTCCCCCAAAAGATG	GCCATTTGGGAACTTCTCAT

### Transcriptomic analysis

For RNA-seq data analysis, clean reads were obtained by removing reads containing adapter, reads containing ploy-N and inferior quality reads from raw data. All the downstream analyses were based on the clean data with high quality. Paired-end clean reads were aligned to the reference genome using Hisat2 (version 2.0.5). StringTie (version 1.3.3b) (22) was used to make new gene prediction, and featureCounts (version 1.5.0-p3) (23) calculated fragments per kilobase of transcript sequence per millions of each gene according to the length of the gene and calculated the reading mapped to the gene. Differential expression analysis of two groups was performed using the DESeq2 R package (version 1.20.0) (24). The *p-*value was adjusted using the Benjamini–Hochberg method. A *p*-value of 0.05 and fold change of 1 were assigned as the threshold for significantly differentially expressed.

To further focus on the important differential genes and regulatory pathways, we first performed the protein interaction networks of differentially expressed genes (DEGs) using the Search Tool for the Retrieval of Interacting Genes/Proteins database 11.0 (25), which knows and predicts protein–protein interactions. Then, the network was introduced Cytoscape (version 3.7.1) for visual editing and found the node genes with degree ≥2. According to the selected node genes, Gene Ontology (GO) enrichment analysis and statistical enrichment of Kyoto Encyclopedia of Genes and Genomes (KEGG) pathway were realized using the package “ClusterProfifiler” (version 3.4.4) (26).

### Statistical evaluation

Data are presented as the mean ± SEM. Two-tailed Student's *t*-tests and rank-sum test (the Newman–Keuls *post-hoc* test) using the SPSS 26.0 software package (IBM, USA) were used for single comparisons. The relationship between different variables was determined by calculating Pearson's or Spearman's correlation coefficients, as appropriate. Statistical significance was assumed if *p* < 0.05.

## Results

### Two percent PS intake increased free sterols levels in multiple tissues

**Figure 1:**
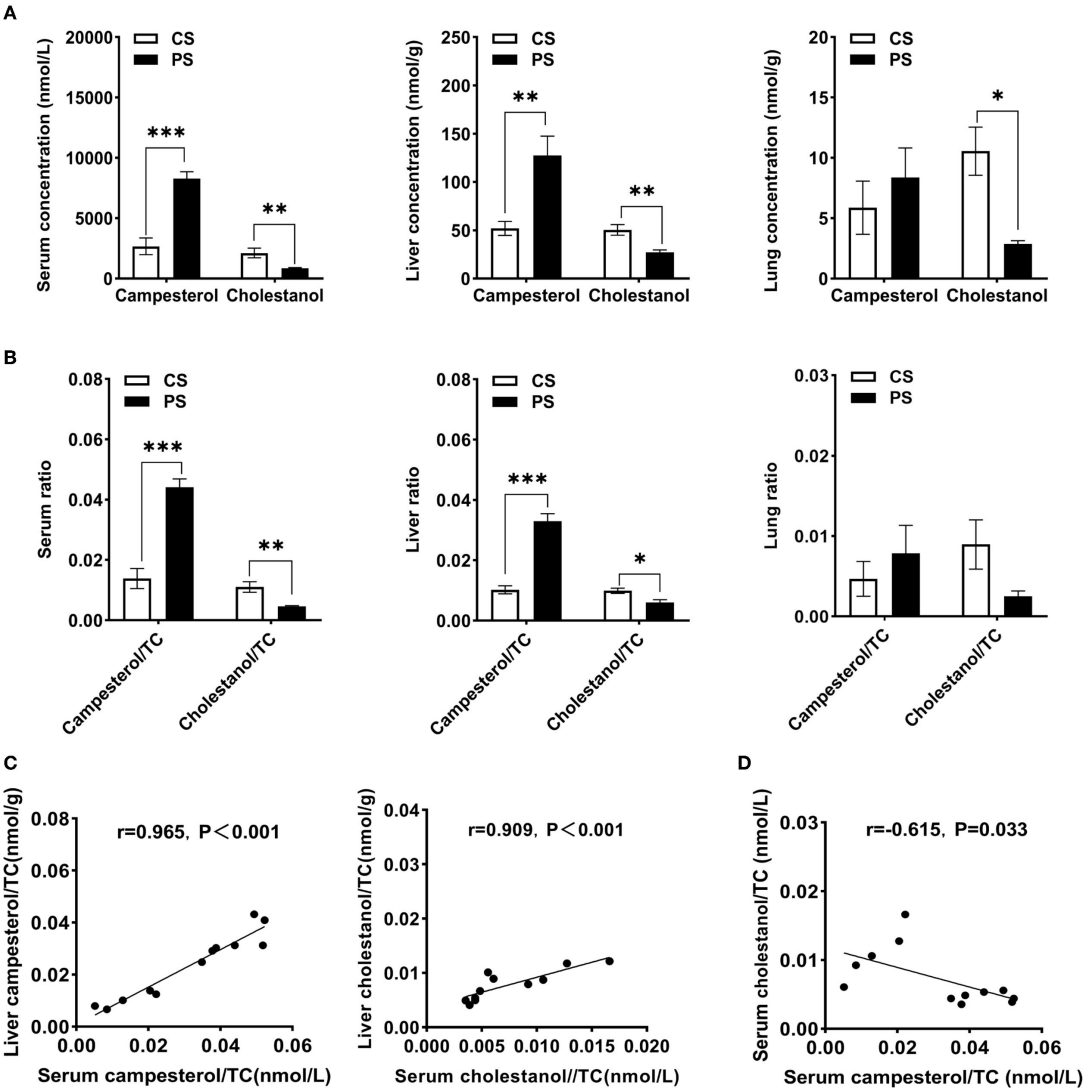
Tissue-free sterols detection. **(A)** Concentrations of campesterol and cholestanol in serum (nmol/L), liver (nmol/g wet weight), and lung (nmol/g wet weight). **(B)** The ratios of campesterol and cholestanol concentrations to cholesterol in the serum, liver, and lung. **(C)** The correlation between hepatic and circulating campesterol/TC or cholestanol/TC. **(D)** The correlation between campesterol/TC and cholestanol/TC in serum. *n* = 5–7 mice/group. Results are shown as the mean ± SEM. *p < 0.05. **p < 0.01, ***p < 0.001.

First, we determined the total concentrations of free sterols in different tissues of mice, including serum, liver, and lung. In line with the previous report (20), the 2% PS diet significantly increased campesterol concentrations and its ratio to cholesterol in the serum and liver of mice compared with the CS group. In contrast, the cholestanol levels and its ratio to cholesterol showed a reduction in the PS group. The same trends were observed in the lung (Figures 1A,B). Hepatic ratios of campesterol and cholestanol to cholesterol levels can increase with the circulating ratio (Figure 1C). A significant negative correlation (*r* = −0.615, *p* = 0.033) between the ratio of cholestanol to cholesterol and the ratio of campesterol to cholesterol in serum was found (Figure 1D).

### Two percent PS intake reduced serum TC level, with the TG level unchanged

**Figure 2:**
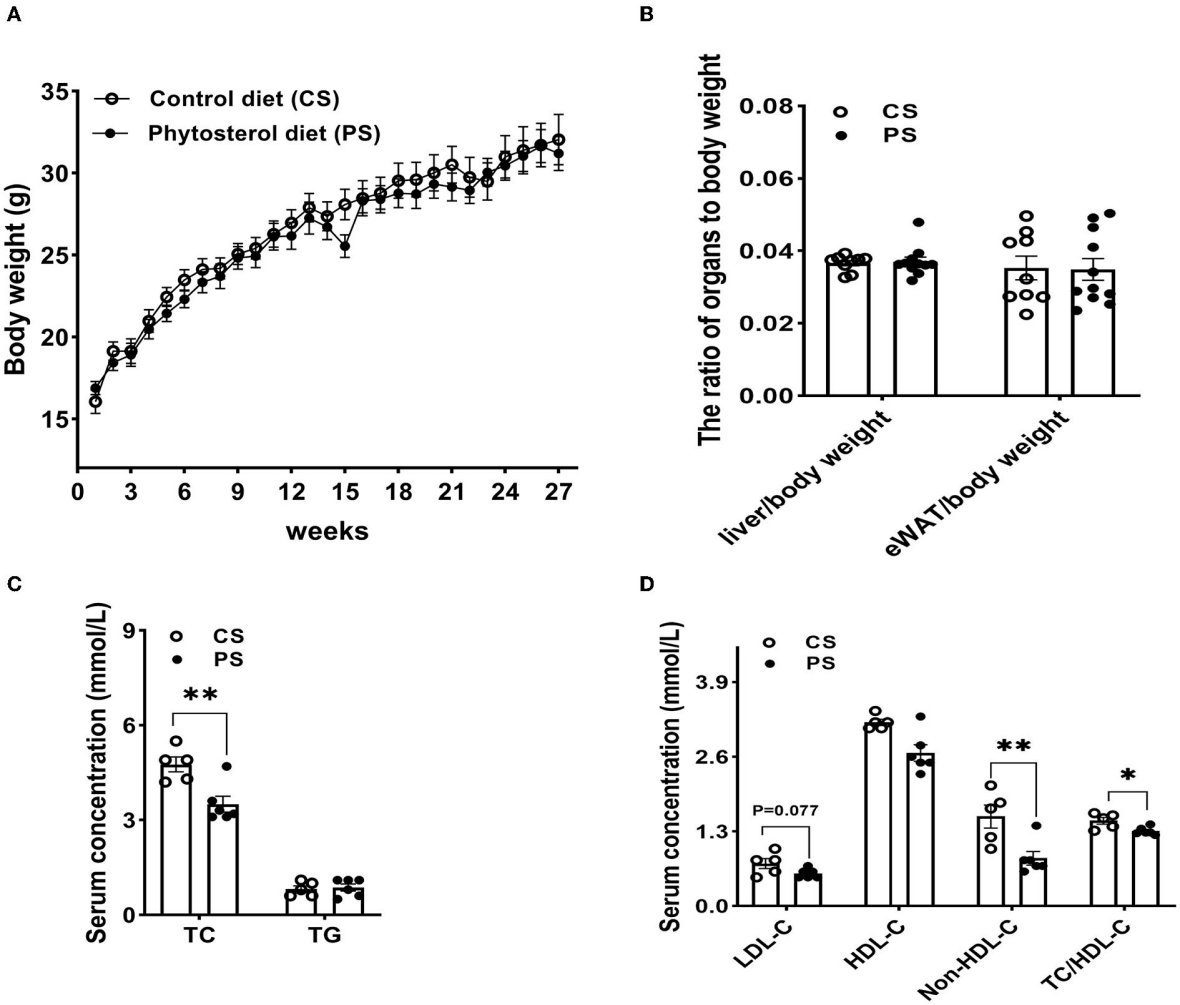
Weight and biochemical indicators. **(A)** Body weight. **(B)** Liver to body weight ratio and eWAT to body weight ratio (*n* = 9–11 mice/group). **(C)** Serum TC and TG levels (*n* = 5–6 mice/group). **(D)** LDL-C, HDL-C, non-HDL-C concentrations and TC/HDL-C (*n* = 5–6 mice/group). Results are the mean ± SEM. *p < 0.05, **p < 0.01.

After 28 weeks of 2% PS diet or CS diet, there was no significant difference in body weight between the two dietary groups, while the overall weight of mice in the PS group was lower (Figure 2A). The liver or epididymal white adipose tissue (eWAT) to body weight ratios did not significantly differ between the two dietary groups (Figure 2B). Compared with the CS group, 2% PS intake significantly reduced the level of serum TC, with TG level unchanged (Figure 2C). Comparison between groups showed that the PS group was better than the CS group in terms of decreasing the levels of serum LDL-C, non-HDL-C, and TC/HDL-C (Figure 2D).

### Liver transcriptome profiles and hepatic pathophysiological changes in response to PS intake

**Figure 3:**
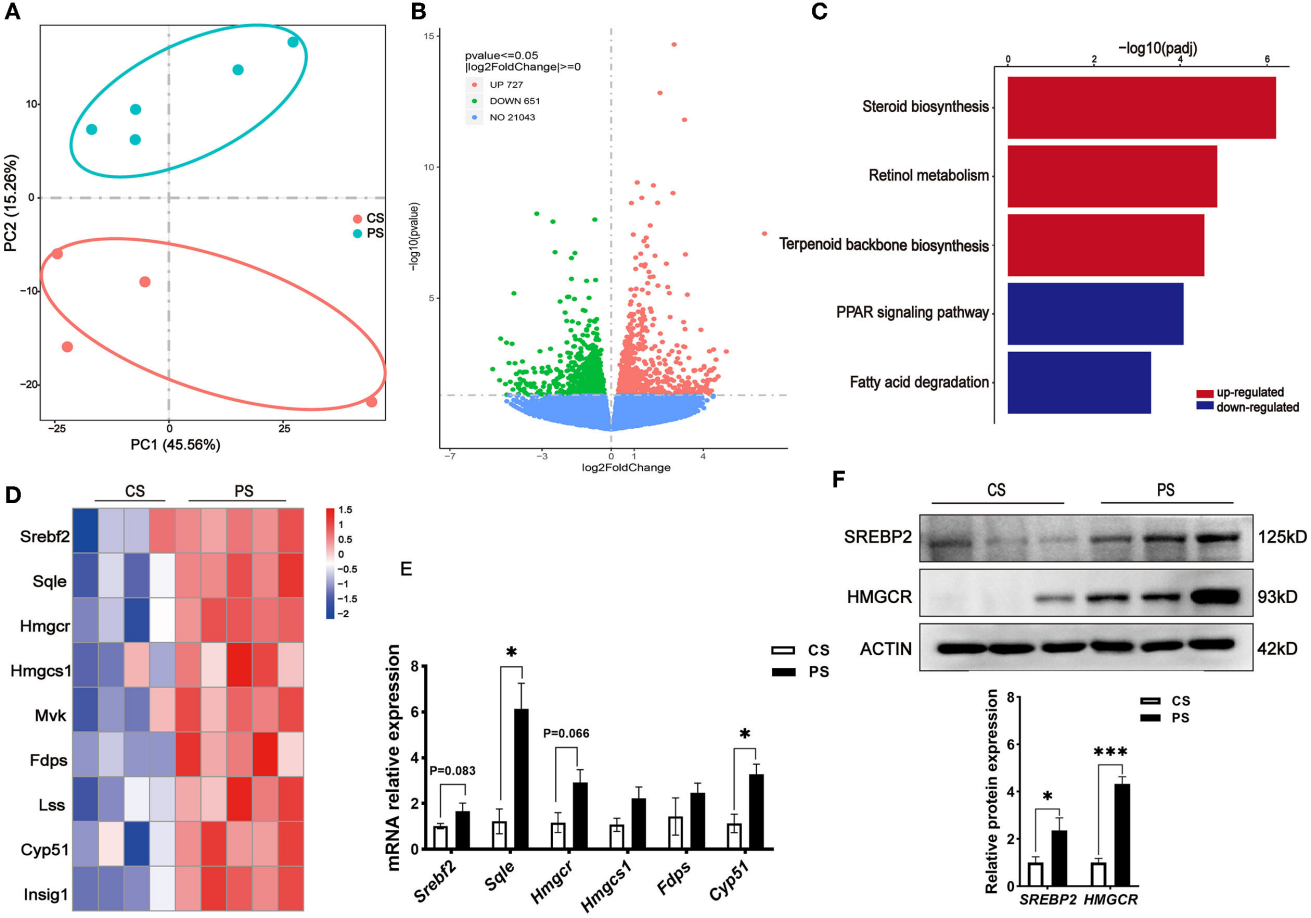
Response of the liver transcriptome to different dietary phytosterol intakes. **(A)** Principal component analysis (PCA) score plots of the whole transcriptomic dataset in the liver. Each dot represents an observation (animal) projected onto the first (horizontal axis) and second (vertical axis) PCA variables. **(B)** Volcano plot of differentially expressed genes in the liver of CS and PS group mice. Colors indicate *p* <0.05 and fold change >1 (red), *p* < 0.05 and fold change <1 (green), and non-significant (blue). **(C)** Representative bar charts of lipid metabolism-related pathways enriched by KEGG analysis in the liver. Red and blue colors indicate upward and downward revisions, respectively. **(D)** Heatmap of genes involved in cholesterol synthesis. **(E)** Validation of gene expression in the liver derived from RT-qPCR assay. **(F)** Western blot analysis of SREBP2 and HMGCR proteins in the liver and the quantitative densitometric analysis normalized against β-actin. *n* = 3 mice/group, the results are presented as the mean ± SEM, representative of three independent experiments, *p < 0.05, ***p < 0.001.

**Figure 4:**
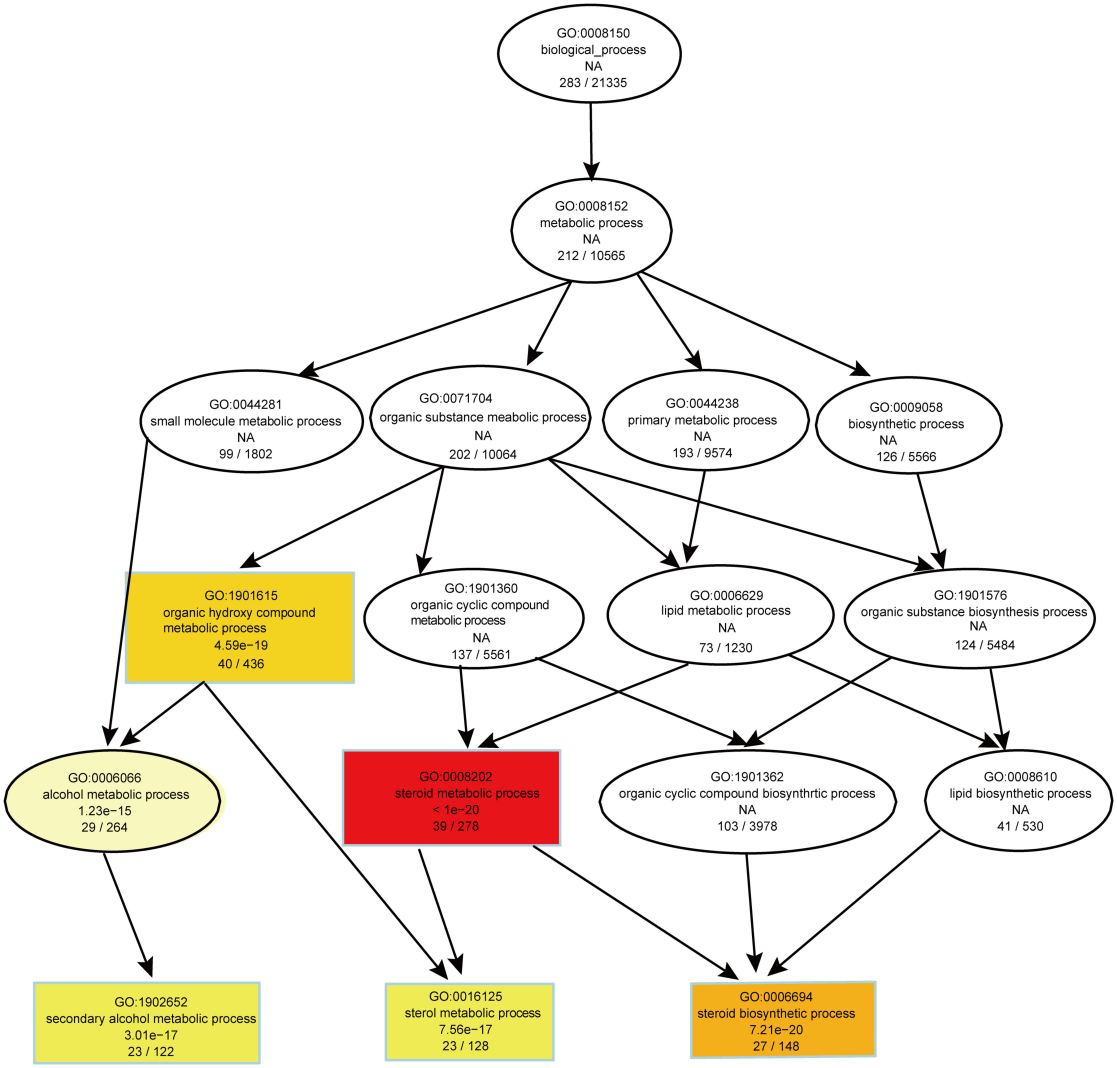
Directed acyclic graph of GO term enrichment analysis for significantly upregulated DEGs in liver of 2% phytosterol-feeding mice.

To evaluate the effects of PS intake on the liver transcriptome, we quantified the gene expression profile by RNA-seq. Principal component analysis (PCA) of the transcriptome revealed that there were significant differences in gene expression in the liver of mice with different dietary patterns (Figure 3A). We recognized 727 upregulated and 651 downregulated genes (Figure 3B). Directed acyclic graph analysis of GO term enrichment analysis showed that significantly upregulated DEGs were enriched to the three biological processes of secondary alcohol metabolism, sterol metabolic, and steroid biosynthetic process in the liver of PS-fed mice (Figure 4), (Supplementary Table S1). KEGG enrichment analysis also identified that steroid biosynthesis and terpenoid backbone biosynthesis were upregulated by comparing the PS group with the CS group (Figure 3C), (Supplementary Table S2). Especially the genes encoding the cholesterol cascade synthesis were expressed at high levels in the PS dietary group (Figure 3D). RT-quantitative PCR (RT-qPCR) and Western blot further confirmed the changes in the expression of several key enzymes encoding cholesterol synthesis in the liver (Figures 3E,F).

**Figure 5:**
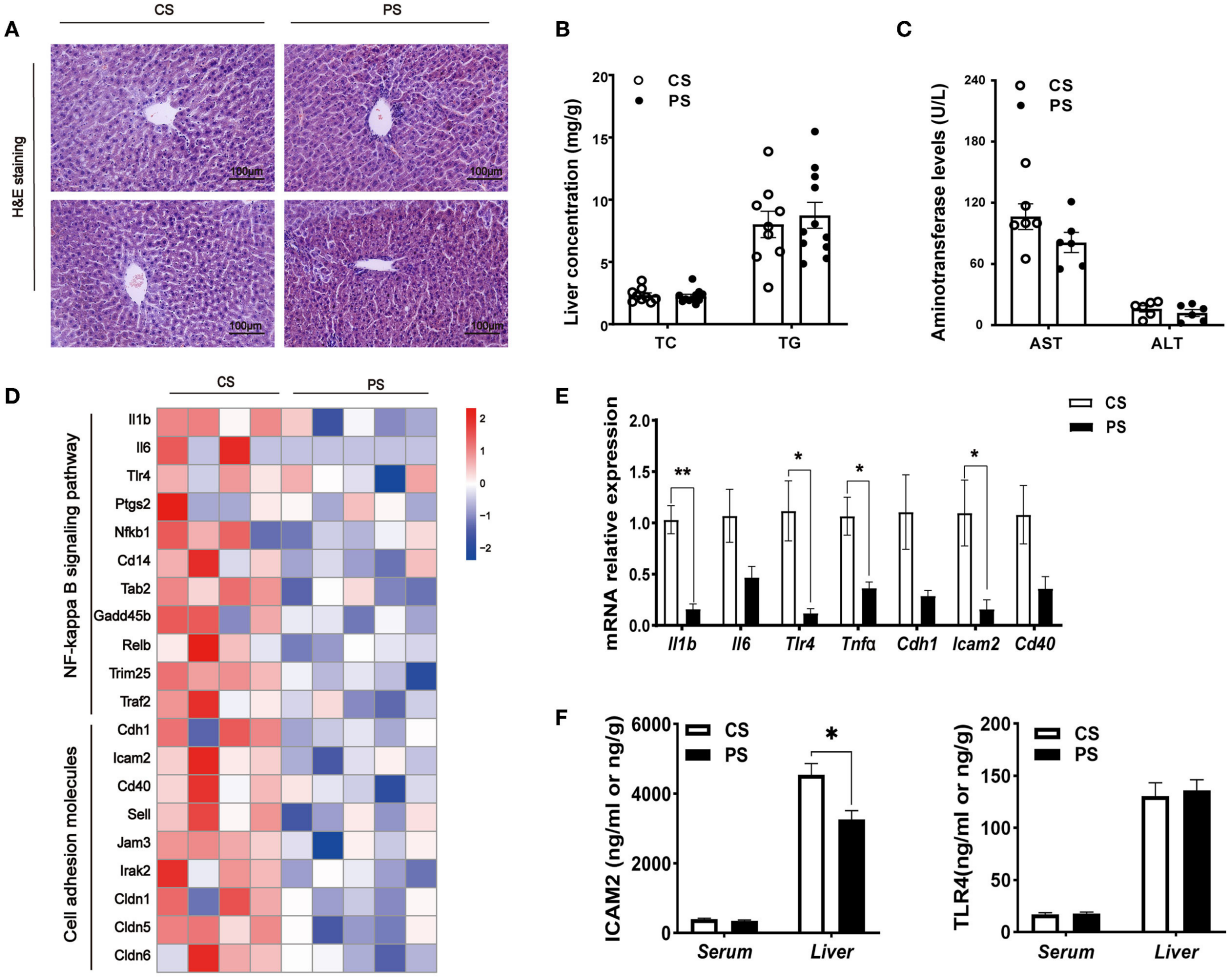
Hepatic pathophysiological changes and inflammation. **(A)** H&E staining of the liver of mice in two groups at the end of the 28th week of the experiment. **(B)** TC and TG concentrations in the liver (*n* = 9–11 mice/group). **(C)** The AST and ALT levels (*n* = 5–7 mice/group). **(D)** Heatmap profiling the expression of genes related to inflammation in the liver (*n* = 3–5 mice/group). **(E)** Validation of gene expression in the liver derived from RT-qPCR assay. **(F)** ELISA kit detecting the concentrations of ICAM2 or TLR4 in the serum and liver (*n* = 4–5 mice/group). The results are presented as the mean ± SEM, representative of three independent experiments, *p < 0.05, **p < 0.01.

We further assessed whether changes in the transcriptome profiles resulted in the accumulation and redistribution of lipid in the liver. After H&E staining, the liver tissue sections of mice were qualitatively observed, and no obvious fat droplet and inflammatory cell infiltration were observed (Figure 5A). The concentrations of TC and TG in the liver of the PS group were not significantly increased (Figure 5B). In addition, no significant difference in AST and ALT between the two dietary groups was found (Figure 5C). At the transcriptome level, we identified that the pathways associated with inflammation were markedly downregulated, including cell adhesion molecules, leukocyte transendothelial migration, and NF-kappa B signaling pathway (Supplementary Table S2). PS diet significantly decreased the level of genes associated with inflammation, such as *Il1b, Tlr4, Tnf*α, and *Icam2* (Figures 5D,E). We further examined the protein levels of pro-inflammatory cytokines in serum and liver, and found that the level of ICAM2 in liver decreased significantly (Figure 5F). Taken together, with the decrease in circulating cholesterol concentration caused by PS diet, the expression of endogenous cholesterol synthesis genes was upregulated in the liver. Healthy PS-fed mice did not promote lipid droplet accumulation or inflammatory response in the liver.

### Lung transcriptome profiles in response to PS intake

**Figure 6:**
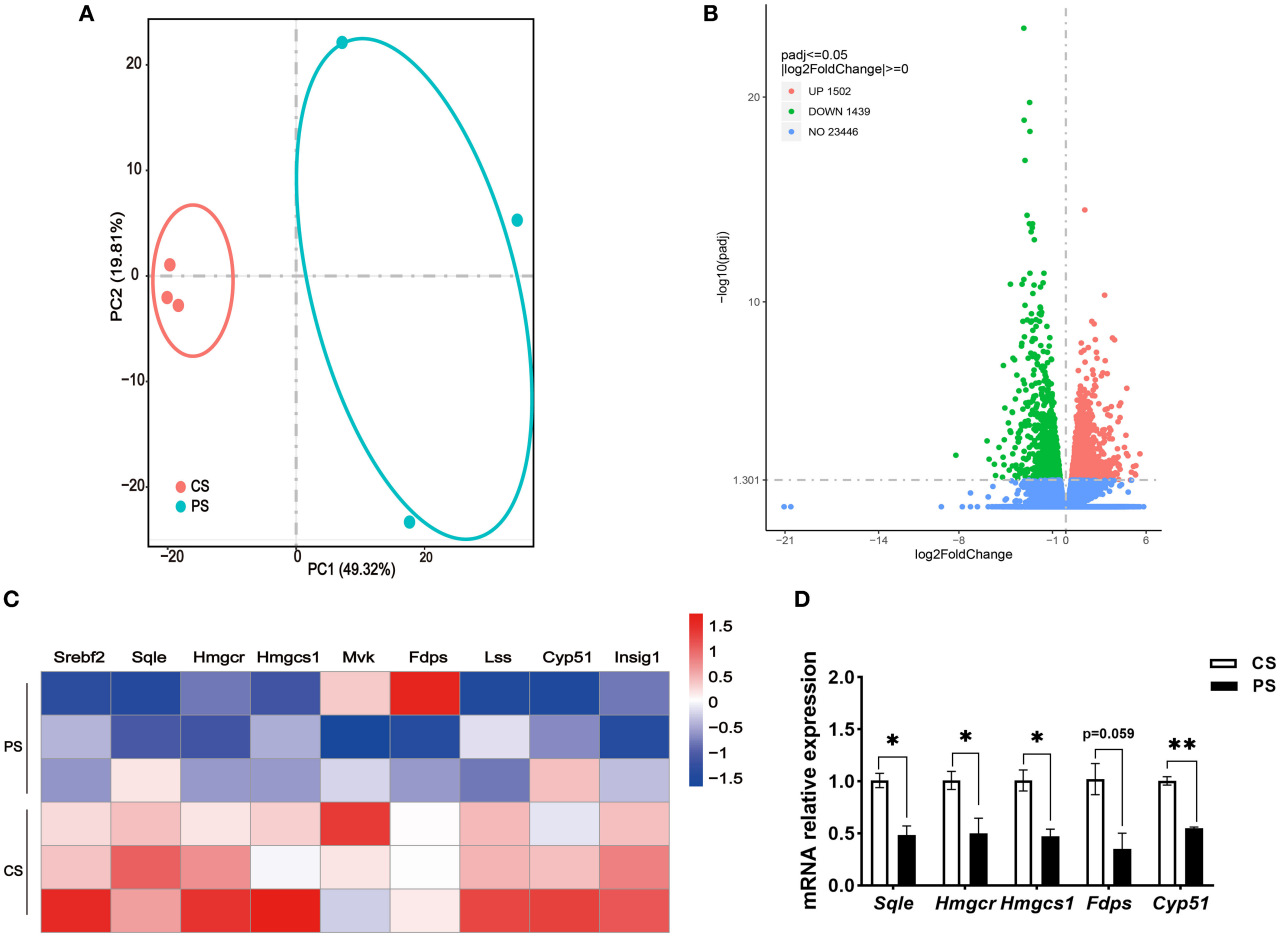
Response of the lung transcriptome to phytosterols intakes. **(A)** Principal component analysis (PCA) score plots of the whole transcriptomic dataset in the lung. Each dot represents an observation (animal) projected onto the first (horizontal axis) and second (vertical axis) PCA variables. **(B)** Volcano plot of differentially expressed genes in the lung of mice in the CS and PS group. Colors indicate *p* < 0.05 and fold change >1 (red), *p* < 0.05 and fold change <1 (green), and nonsignificant (blue). **(C)** Heatmap of genes involved in cholesterol synthesis in the lung of two dietary group mice. **(D)** Validation of gene expression derived from RT-qPCR assay. *n* = 3 mice/group, the results are presented as the mean ± SEM, representative of three independent experiments, *p < 0.05, **p < 0.01.

**Figure 7:**
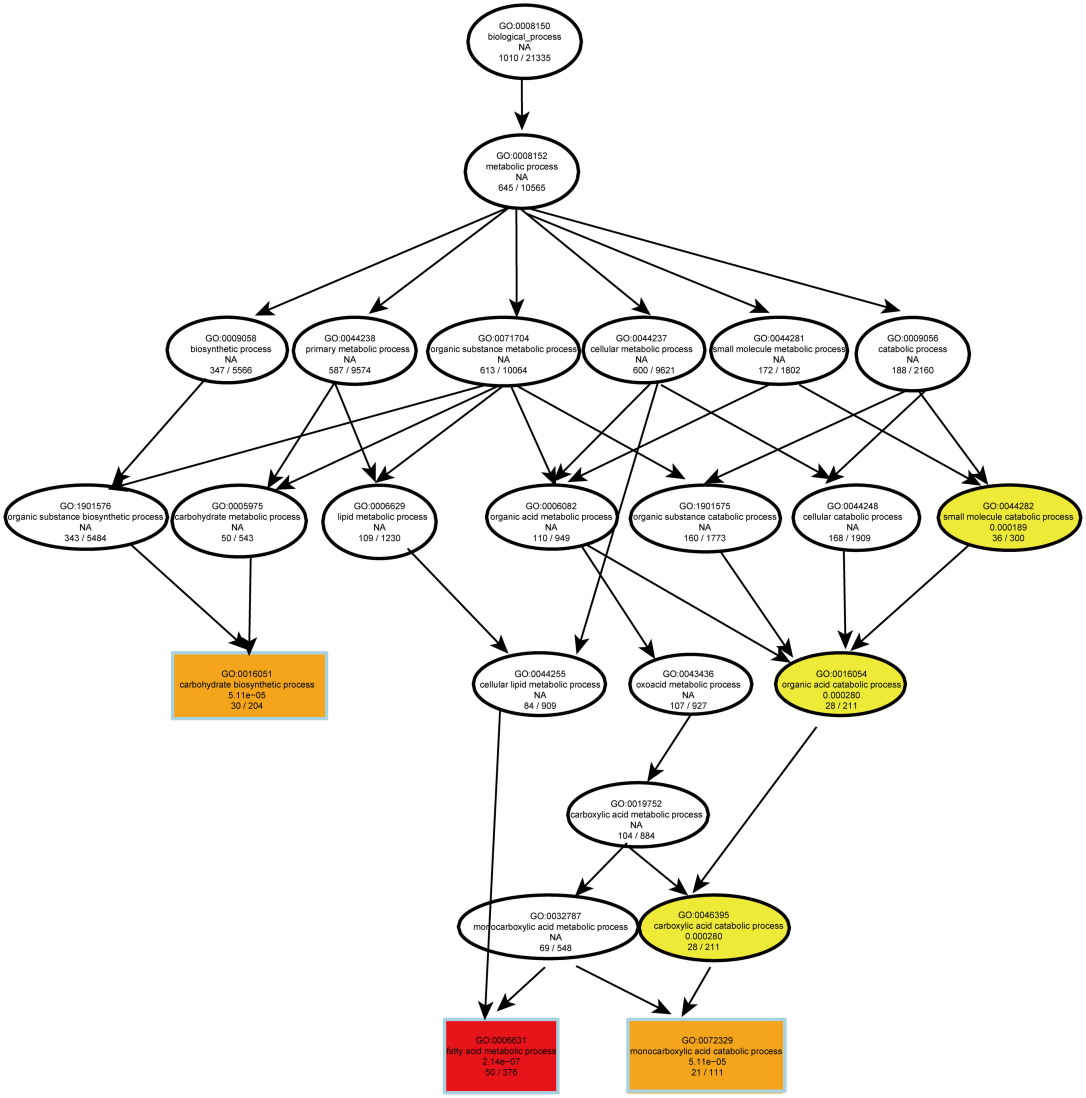
Directed acyclic graph of GO term enrichment analysis for significantly downregulated DEGs in the lung of 2% phytosterol-feeding mice.

**Figure 8:**
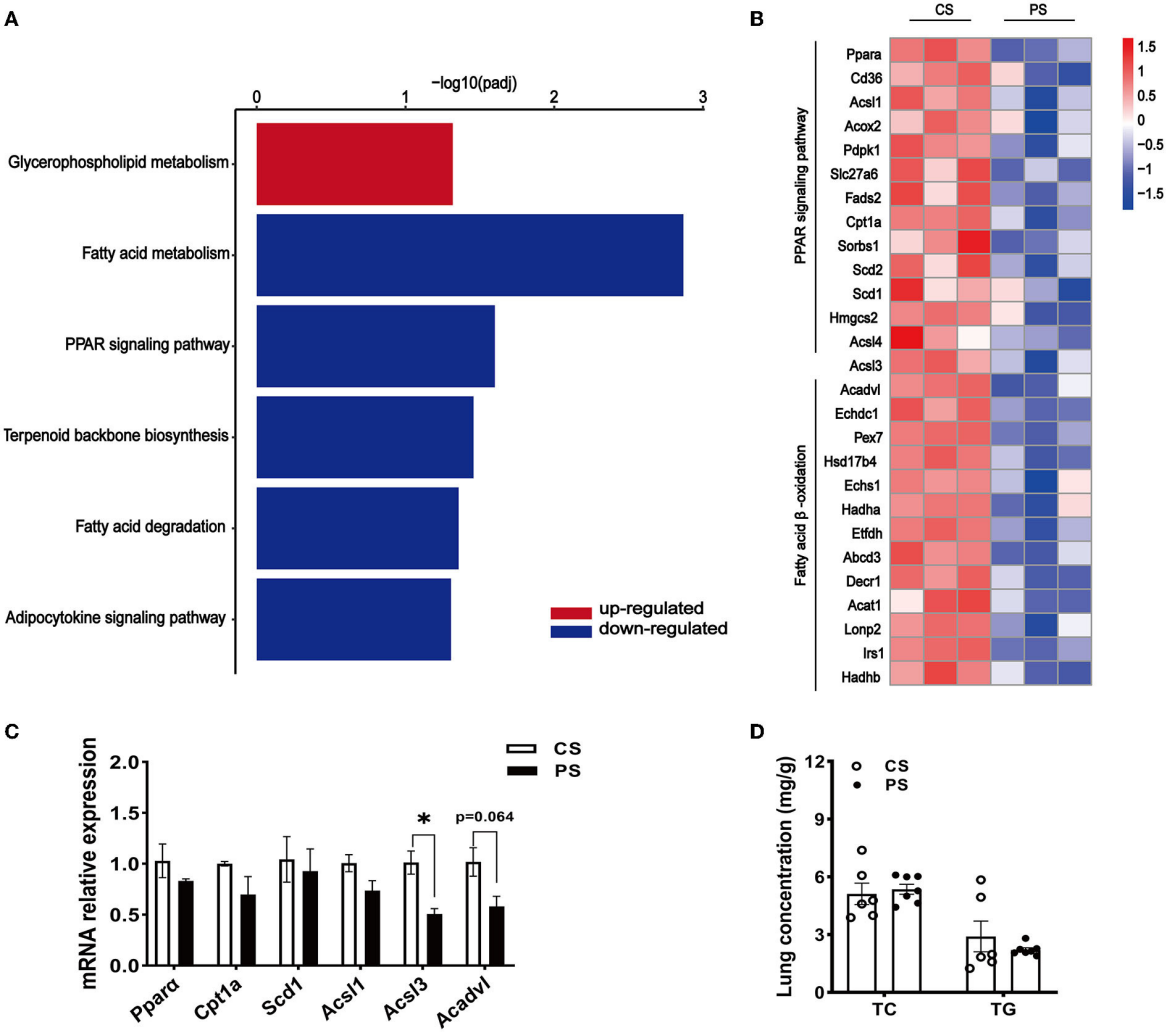
Changes in fatty acid metabolism transcription profile in the lung of PS diet-fed mice. **(A)** Representative bar charts of lipid metabolism-related pathways enriched by KEGG analysis in the lung. Red and blue colors indicate upward and downward revisions, respectively. **(B)** Heatmap profiling the gene expression of genes related to fatty acid transport and oxidation in the lung. **(C)** Validation of gene expression in the lung derived from RT-qPCR assay. **(D)** TC and TG concentrations in the lung. *n* = 3–7 mice/group, the results are presented as the mean ± SEM, representative of three independent experiments, *p < 0.05.

The RNA-seq analysis taken in the lung showed that the gene expression profiles were different between the two dietary groups (Figure 6A). Compared with CS diet-fed mice, we identified 1,502 upregulated and 1,439 downregulated genes in the lung of PS-fed mice (Figure 6B). In contrast to the liver's response to PS, the expression of genes encoding the cholesterol synthesis was decreased in the lung (Figure 6C). The results of RT-qPCR showed that the mRNA levels of *Hmgcr, Hmgcs1, Sqle*, and *Cyp51* were decreased (Figure 6D). In addition, directed acyclic graph analysis of GO term enrichment analysis pointed out that downregulated DEGs were enriched to fatty acid metabolic process in the lung of PS-fed mice (Figure 7), (Supplementary Table S3). KEGG enrichment analysis also identified that fatty acid metabolism, PPAR signaling pathway, fatty acid degradation, and adipocytokine signaling pathway were downregulated (Figure 8A), (Supplementary Table S4). The expression of genes associated with fatty acid transport and oxidation was obviously downregulated in the lung of the mice in the PS group (Figure 8B). The RT-qPCR results of *Ppar*α, *Acsl3*, and *Acadvl*, which are classically recognized to be associated with fatty acid transport and oxidation, were roughly the same as those of the transcriptome (Figure 8C). However, there were no significant differences in total TC and TG levels in lung tissues between the two groups (Figure 8D). Anyway, these data may suggest the specific role of PS in lung lipid metabolism.

## Discussion

With new evidence indicating that PS could be taken up into the circulation and may have effects beyond lowering LDL-cholesterol, fundamental studies are needed to figure out the systemic and tissue-specific pathophysiological effect of long-term PS feeding. In this study, we demonstrated that 28 weeks of 2% PS feeding effectively reduced circulating TC level in the healthy C57BL/6J mice. In the liver, the lipid metabolic central organ, the expressions of hepatic endogenous cholesterol synthesis genes were upregulated, but with no hepatic lipid accumulation observed. Meanwhile, inflammatory pathways were downregulated in the liver. In peripheral tissues like the lung, genes associated with cholesterol synthesis were downregulated, together with fatty acid transport and oxidation genes downregulated.

Within the reference intake range for the general population, the circulating concentration of most free PS maintains dynamic equilibrium, but a higher intake can increase PS levels in different tissues. Compared with the recommended 2 g of PS daily supplementation in human health promotion, the widely used dosage of 2% PS formula in mice studies is pretty high (18, 27). The serum campesterol level we detected in this study was in micromoles, which was comparable to the circulating levels of plant sterols (7–24 μmol/l) in humans (4). Consistent with previous reports (4, 28–30), the elevated campesterol concentrations and their ratio to cholesterol were also presented in liver and lung. Meanwhile, the decreased concentration of cholestanol and its ratio to cholesterol indirectly indicated the cholesterol absorption reduction. As a result, significantly lowered circulating TC and non-HDL concentration were presented in the 2% PS-treated mice. In response to the decreased circulating cholesterol level, the compensated endogenous cholesterol synthesis was increased in the liver, which was manifested by the significantly upregulated genes related to the synthesis of cholesterol cascade products such as *Srebf2, Hmgcr, Hmgcs1*, and *Sqle*. Consistently, previous research using sterol balance techniques observed that dietary use of sitostanol ester margarine reduced absorption, increased fecal elimination, and stimulated compensatory synthesis of cholesterol in human beings (31). Our transcriptome data gave more profound data regarding the important role of the liver in compensatory regulating of cholesterol homeostasis in the body.

To test whether the triggered feedback mechanism and the increased hepatic concentration of PS could cause any liver damage, we observed the histopathological features of the liver. The 28-week supplementation of PS to the diet in healthy mice neither caused lipid droplet accumulation and inflammatory cell infiltration in the liver tissues, nor did it affect the levels of TC and TG in the liver. Meanwhile, the levels of liver enzymes like AST or ALT, and the expression of key genes of inflammation were not significantly promoted by PS diet. This is consistent with the earlier observation by Gregory Guthrie (32) that PS alone does not directly induce inflammatory responses in hepatocytes. The hepatobiliary injury caused by PN using soybean oil lipid emulsions might depend on the route of administration (intravenous drip), and dietary plant sterols that are absorbed from the diet, incorporated into chylomicron and transported to the liver do not cause hepatic injury (33).

Emerging evidence has demonstrated that the dynamic balance of cholesterol and lipoprotein is important for lung physiology. The most compelling evidence was the pathological pulmonary phenotypes observed in the mice with genetic ablation of apoA-I (34), apoE (35), ATP binding cassette subfamily A member 1 (ABCA1) (36), and ATP binding cassette subfamily G member 1 (ABCG1) (11, 37) genes. Without those specific efflux receptors, lipid would overload lung resident cells and impair the function of alveolar surfactants (38, 39). Furthermore, a recent study has found that β-sitosterol could alleviate chronic alveolar epithelial injury through suppression of epithelial–mesenchymal transformation by inhibiting the TGF-β/Snail pathway (40). Our data showed that unlike the compensatory endogenous cholesterol synthesis in the liver, the expression of genes related to cholesterol synthesis was downregulated in the lung of the PS-supplemented diet-fed mice, together with pathways relating fatty acid synthesis, transport, and oxidation downregulated. Since evidence is accumulating regarding several intracellular metabolic pathways, including cholesterol synthesis, fatty acid oxidation, and glycolysis tightly regulate immune cell function (41), and PS exhibit anti-inflammatory action *via* different modes (42), studies relating lipid metabolism to the physiopathology of lung diseases is promising in the near future.

## Conclusion

A total of 28 weeks of 2% PS diet could effectively reduce the circulating TC levels in healthy C57BL/6J mice. The liver persists protective effect to avoid uncontrolled decline in circulating cholesterol level by balancing increasing hepatic cholesterol synthesis, thus causing no obvious hepatic lipid accumulation and inflammation. The mechanisms and effects related to diminished cholesterol synthesis, fatty acid transport, and oxidation in the lung with long-term PS supplementation need to be further elucidated.

## Data availability statement

The datasets presented in this study can be found in online repositories. The names of the repository/repositories and accession number(s) can be found in the article/Supplementary material.

## Ethics statement

The animal study was reviewed and approved by the Biomedical Ethics Committee of Chongqing Medical University (Chongqing, China).

## Author contributions

XL: conceptualization and methodology. QZ and JW: experimental conduction and data analysis. DH, JL, and SW: data curation. QZ: figure organization and writing—original draft preparation. XL: writing—review and editing, supervision, and project administration. All authors contributed to the article and approved the submitted version.

## Funding

The study was funded by the Yihaikerry Nutrition and Food Safety Foundation, Chinese Nutrition Society (CNS-W2018A45); the project of Technology Innovation and Application, Chongqing, China (cstc2019jscx-msxmX0280); and the project of the top-notch talent cultivation program for the graduate students of Chongqing Medical University (BJRC202029).

## Conflict of interest

The authors declare that the research was conducted in the absence of any commercial or financial relationships that could be construed as a potential conflict of interest.

## Publisher's note

All claims expressed in this article are solely those of the authors and do not necessarily represent those of their affiliated organizations, or those of the publisher, the editors and the reviewers. Any product that may be evaluated in this article, or claim that may be made by its manufacturer, is not guaranteed or endorsed by the publisher.

## Abbreviations

ABCA1: ATP binding cassette subfamily A member 1
ABCG1: ATP binding cassette subfamily G member 1
ALT: alanine aminotransferase
AST: aspartate aminotransferase
DEG: differentially expressed genes
ELISA: enzyme-linked immunosorbent assay
eWAT: epididymal white adipose tissue
GO: Gene Ontology
HDL-C: high-density lipoprotein cholesterol
KEGG: Kyoto Encyclopedia of Genes and Genomes
LDL-C: low-density lipoprotein cholesterol
PCA: principal component analysis
TC: total cholesterol
TG: triglyceride
UPLC-MS: ultra-performance liquid chromatography-mass spectrometry.
